# Improving human papillomavirus (HPV) testing in the cervical cancer elimination era: The 2021 HPV LabNet international proficiency study

**DOI:** 10.1016/j.jcv.2022.105237

**Published:** 2022-09

**Authors:** Laila Sara Arroyo Mühr, Carina Eklund, Camilla Lagheden, Ola Forslund, Karin Dahlin Robertsson, Joakim Dillner

**Affiliations:** a Department of Laboratory Medicine, Karolinska Institutet, Stockholm, 141 86, Sweden; b Department of Laboratory Medicine, Division of Medical Microbiology, Lund University, Lund, 221 85, Sweden; c EQUALIS AB, Uppsala, 751 09, Sweden; d Center for Cervical Cancer Prevention, Karolinska University Hospital, Stockholm, 141 86, Sweden

**Keywords:** Human papillomavirus, Quality assurance, International standards, Cervical cancer, Cancer eradication

## Abstract

**Background:**

Proficient Human Papillomavirus (HPV) genotyping services are essential to support HPV and cervical cancer elimination strategies, in particular to support HPV vaccine research.

**Objectives:**

To perform a global HPV genotyping proficiency study, with evaluation in relation to previous proficiency studies.

**Study design:**

The proficiency panel contained 44 coded samples (40 samples containing one or more purified HPV types (HPV6/11/16/18/31/33/35/39/45/51/52/56/58/59/68a/68b) in human DNA, 1 human DNA control and 3 DNA extraction controls). Proficiency required detection of both single and multiple infections of 50 International Units of HPV 16/18, of 500 genome equivalents for other HPV types and no false positivity.

**Results:**

One hundred and thirty-two laboratories submitted 211 datasets. Most assays used (182/211 datasets) were commercially available. An all-time high of 75% of the datasets were 100% proficient. One or more false positives were found in 17.5% of datasets. Among laboratories who participated in the 2019 proficiency study, full proficiency increased from 25% in 2019 to 60% in 2021. The high overall proficiency was mostly attributable to a large number of new laboratories, which used similar assays.

**Conclusions:**

The worldwide deterioration in comparability and reliability of HPV testing found in 2019 is now reversed and an overall increase in proficiency is found.

## Highlights

• The 2021 global HPV genotyping proficiency study included 132 laboratories.
• An all-time high proportion of datasets were fully proficient: 75%.
• High proficiency detected among many laboratories without previous participation.
• Laboratories with previous proficiency study participation also improved.

**Abbreviations**
AFRO: African Regional Office
EMRO: Eastern Mediterranean Regional Office
EURO: European Regional Office
Equalis: External quality assessment of laboratory medicine in Sweden
GE: Genome equivalents
HPV: Human papillomavirus
International HPV Reference Center: IHRC
IU: International units
LabNet: Laboratory Network
PAHO: Pan American Health Organization
PP: Proficiency panel
SEARO: South-East Asian Regional Office
WHO: World Health Organization
WPRO: Western Pacific Regional Office.

## 1 Introduction

In the era of cervical cancer elimination, many laboratories perform Human Papillomavirus (HPV) detection and the number of different assays used has increased exponentially. [1] In 2012 there were already 125 different commercial HPV tests on the global market and in 2020, there were 254 distinct HPV assays and 425 assay variants. [2, 3]

Accurate and internationally comparable HPV detection is a must for both research and evaluation of HPV vaccination, as well as for screening programs. [4] A recent meta-review of publications validating the different HPV assays found that only 11 HPV DNA assays had published in the peer-reviewed literature with analytical/clinical evaluation. [3]

The International HPV Reference Center (IHRC) coordinated the Global WHO HPV Laboratory Network at the outset and has since 2008 coordinated global HPV genotyping proficiency studies to a) assess the proficiency of HPV typing assays when routinely used in laboratories worldwide, b) evaluate the sensitivity and type-specificity of HPV detection of the different HPV assays when routinely used in laboratories worldwide and, c) identify problems with any assays routinely used. [5], [6], [7], [8], [9], [10]

The proficiency panels (PPs) consist of blinded samples with defined amounts of HPV DNA (traceable to International Standards) in a background of human DNA, as well as extraction controls. Laboratories genotype these samples with their standard methods.

In 2019 we found a global decrease in proficiency of HPV genotyping [10] and have therefore increased the ambition level of our international quality assurance program, including issuing of PPs every year. The present report describes the results of the 2021 proficiency study.

## 2 Material and methods

### 2.1 2021 proficiency panel (PP)

The 2021 PP included 44 coded samples (40 samples containing one or more purified HPV plasmids diluted in TE buffer (10 mM TRIS-HCl, 0.1 mM EDTA, pH 8.0) with 10 ng/ul of human placental DNA (Sigma-Aldrich no 7011), 1 negative control (TE buffer with 10 ng/µl human placenta DNA) and 3 samples of cell lines used as controls for the DNA extraction step in the testing (Table 1).

**Table 1:** 2021 Proficiency panel composition and percentage of laboratories reporting correct HPV type, with no false positive HPV type detected.

HPV types	HPV IU or genome equivalents per 5 µl	Percent correct data sets^a^ (N)
16	50	96.7 (204 / 211)
16	5	90.5 (191 / 211)
18	50	98.1 (207 / 211)
18	5	89.1 (188 / 211)
6	500	95.6 (152 / 159)
6	50	93.7 (149 / 159)
11	500	98.7 (157 / 159)
11	50	93.7 (149/ 159)
31	500	99.0 (208 / 210)
31	50	91.4 (192 / 210)
33	500	97.6 (205 / 210)
33	50	94.8 (199 / 210)
35	500	97.6 (205 / 210)
35	50	95.7 (201 / 210)
39	500	97.6 (205 / 210)
39	50	93.3 (196 / 210)
45	500	98.1 (207 / 211)
45	50	96.7 (204 / 211)
51	500	98.6 (206 / 209)
51	50	91.4 (191 / 209)
52	500	96.7 (203 / 210)
52	50	94.8 (199/ 210)
56	500	95.2 (198/ 208)
56	50	89.4 (186 / 208)
58	500	97.1 (204 / 210)
58	50	93.3 (196 / 210)
59	500	98.6 (206 / 209)
59	50	96.2 (201 / 209)
68a	500	78.9 (157 / 199)
68a	50	75.9 (151 / 199)
68b	500	92.7 (190 / 205)
68b	50	87.3 (179 / 205)
6, 31, 45, 52	500	95.3 (201 / 211)
6, 31, 45, 52	50	88.6 (187 / 211)
11, 33, 51, 58	500	91.9 (194 / 211)
11, 33, 51, 58	50	91.5 (193 / 211)
16, 56, 59, 68^a,b^	500	93.4 (197 / 211)
16, 56, 59, 68^a,b^	50	89.1 (188 / 211)
18, 35, 39, 68^b^	500	93.4 (197 / 211)
18, 35, 39, 68^b^	50	89.1 (188 / 211)
TE buffer with 10 ng/µl human placenta DNA	0	97.2 (205/ 211)
HPV 16 positive SiHa cells	2500	97.6 (204 / 209)
HPV 16 positive SiHa cells	25	94.7 (198 / 209)
HPV-negative C33A cells	0	97.1 (203 / 209)

^a^Denominator may be different within the coded samples as some assays did not claim to detect all HPVs included in the proficiency panel. Evaluation was performed considering only the HPV types that the typing method targeted for. For assays reporting results as an aggregate (e.g. “other genotypes"), proficiency was considered as long as the assay detected the HPV types targeted by the method.

^b^Data sets known not to detect the HPV 68a plasmid in this panel are considered as correct when the other HPV types in the sample are detected

Composition of the samples included in the global HPV DNA genotyping proficiency panel 2021. Proportion of proficient datasets are shown in the last column. Concentrations are given as international units (IU) for HPV 16 and 18 and as genome equivalents (GE) for the other HPV types. Samples with less than 80% correct datasets are highlighted in gray.

The HPV types included were 6, 11, 16, 18, 31, 33, 35, 39, 45, 51, 52, 56, 58, 59, 68a (HPV 68 prototype) and 68b (ME 180 isolate) at different concentrations (50 and 5 international units (IU)/5 ul for HPV 16 and 18, and 500 and 50 HPV genome equivalents (GE)/5ul for the other HPV types). All corresponding proprietors had given approval for the HPV types to be included in the PP.

The PP was pre-tested for validation purposes at the Reference Laboratory in Sweden using a modified GP5+/6+ PCR followed by Luminex-based typing and distributed afterwards to laboratories throughout the world. Distribution of panels in November 2021 was performed by Equalis (External quality assessment of laboratory medicine in Sweden, https://www.equalis.se/en/), following the call for participation and requests received. [10]

The fee for participation was 1000 Euros for commercial entities and 500 Euros for academic and public health entities. Participants from low and lower-middle income countries could apply for waiving of the fee.

All participants were to determine the genotypes in the panel using their standard assays, using their standard sample input volume. There were no particular demands on the assays used and both commercial assays and in-house assays were allowed. In case a participant used several different HPV detection methods, one PP could be used by each assay and one dataset provided from each method.

### 2.2 Data analysis

Datasets from laboratories submitted until the 28th of February 2022 were accepted and compiled by Equalis and analysed by the IHRC.

Proficiency criteria was established by a WHO consensus meeting 14 years ago, [4] requiring detection of at least 50 IU/5ul of HPV 16 and HPV 18 and 500 GE/5 ul of the other HPV types, in both single and multiple infection. For proficiency, it was also required that no false positive HPV type was detected.

Input with 10 or 15 ul was classified as the same IU/GE content as compared to 5 ul input. The panel also included low concentration samples (5 IU/5 ul for HPV16/18 and 50 GE/5 ul for non- 16/18 types) that were intended for educational purposes, training and for providing information on whether the test exceeded the requirements needed for proficient HPV typing. Detection of these low amounts was not required for proficiency.

For assays that did not claim to detect all HPVs included in the PP, evaluation was performed considering only the HPV types that the assay reportedly detected. Consequently, the denominator varies (not all assays targeted all HPV types). For assays reporting results only as aggregated types (e.g. “other"), proficiency was considered as long as the assay detected the HPV types targeted by the method. (e.g.: if an assay only claiming to detect “Other HPV” tested the sample containing HPV 11/33/51/68a and reported that it contained “Other HPV”, it was considered proficient).

The results were sent to all participating laboratories that had paid the fee before February 2022.

## 3 Results

The 2021 proficiency study was distributed to 144 subscribing laboratories. A total of 132 laboratories returned 211 datasets (every laboratory could submit more than one dataset if several different HPV typing methods were used – one dataset per method). Ninety-six laboratories submitted 1 dataset, 13 laboratories submitted 2 datasets, 4 laboratories submitted 3 datasets, 18 laboratories submitted 4 datasets and 1 laboratory submitted 5 datasets.

There were 126 participating laboratories that provided data on type of laboratory. There were 83 diagnostic and screening laboratories, 26 laboratories performing HPV surveillance, 8 clinical trial laboratories and 9 diagnostic test manufacturers. The annual number of samples analysed for HPV per laboratory varied from 100 to > 100,000 per year, with 15 laboratories performing 10,000 tests or more.

Among datasets that typed for at least one HPV type 158/211 (75%) were 100% proficient for the types claimed to be detected by the test. Of these, 119/158 datasets not only correctly identified the content of all required samples, but also correctly identified the content of the training samples that contained amounts that were lower than required for proficiency (samples that contained only 5IU of HPV16/18 and 50GE of other HPVs). Tests that did not type for all the types in the panel could still be 100% proficient, as the denominator was the number of types claimed to be detected by the test (not the number of types included in the panel).

### 3.1 Proficiency by HPV type

The number of laboratories reporting correct HPV types, with no false positivity, are shown in Table 1. Median percent of 100% proficient datasets for the different coded samples was 94.8% (minimum 75.9%, maximum 99.0%). All datasets showed >90% proficiency for all coded samples containing 50 UI or 500 GE/ 5ul except for HPV 68a, where 75.9% and 78.9% of the datasets had detected HPV 68a at 50 GE/5 ul and 500 GE/5 ul, respectively.

### 3.2 Proficiency by WHO region

Laboratories submitting datasets belonged to 6 WHO regions, with Western Pacific Regional Office (WPRO) showing the highest number of participating laboratories (*n* = 59), followed by European Regional Office (EURO, *n* = 49), Pan American Health Organization (PAHO, *n* = 10), African Regional Office (AFRO, *n* = 7), Eastern Mediterranean Regional Office (EMRO, *n* = 5) and South-East Asian Regional Office (SEARO, *n* = 2).

**Fig. 1:**
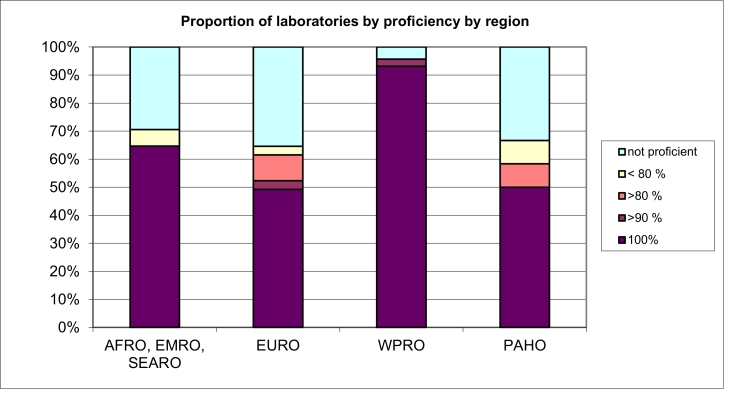
Proficiency for HPV DNA typing by WHO region. Proficiency criteria were: i) detection at least 50 international units (IU) per 5 ul of HPV 16 and HPV 18, in both single and multiple HPV infections, ii) detection of at least 500 genome equivalents (GE) in 5 ul of the other HPV types (not HPV 16 nor HPV 18) in both single and multiple HPV infections and iii) no false positivity detection. AFRO: African Regional Office, EMRO: Eastern Mediterranean Regional Office, EURO: European Regional Office, PAHO: Pan American Health Organization, SEARO: South-East Asian Regional Office, WPRO: Western Pacific Regional Office.

The proportion of laboratory proficiency including all datasets grouped by WHO region is shown in Fig. 1 and Table 2. WPRO showed the highest number of proficient laboratories (93%) and EURO was the region with the highest number of non-proficient laboratories, with only 49% of laboratories being 100% proficient.

**Table 2:** Proportion of datasets submitted by WHO region with ≥90% proficient HPV typing results.

Region (datasets)	Proportion of laboratories with 100% correct typing	Proportion of laboratories with ≥90% correct typing
EURO (65)	49%	52%
AFRO, EMRO, SEARO (17)	65%	65%
PAHO (12)	50%	50%
WPRO (117)	93%	96%

AFRO: African Regional Office, EMRO: Eastern Mediterranean Regional Office, EURO: European Regional Office, PAHO: Pan American Health Organization, SEARO: South-East Asian Regional Office, WPRO: Western Pacific Regional Office.

### 3.3 Proficiency by assay used

**Fig. 2:**
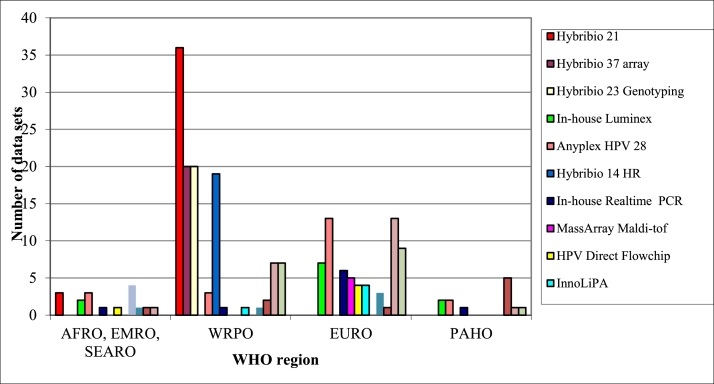
Type of assay in use for HPV DNA typing by WHO region. AFRO: African Regional Office, EMRO: Eastern Mediterranean Regional Office, EURO: European Regional Office, PAHO: Pan American Health Organization, SEARO: South-East Asian Regional Office, WPRO: Western Pacific Regional Office. Data for AFRO, EMRO and SEARO region are combined.

The different assays used are displayed in Fig. 2 and the number of proficient datasets, false positivity, and HPV targets per assay in Table 3.

**Table 3:** Assays used for testing and typing of HPV, HPV region targeted, proficiency and false positivity.

HPV assay type	No. of datasets	HPV region targeted (primers)	No. of proficient data sets					No. of false positive samples per data set				
			100% proficient	99–90% proficient	89–80% proficient	<80% proficient	Not proficient	0 samples	1 sample	2 samples	3 samples	> 3 samples
All assays	211	L1/L2/E1/E2/E4/E5/E6/E7	158	5	7	4	37	174	19	7	1	10
Hybribio 21 array HPV (Hybribio)	39	L1 (MY09/11)	38	0	0	0	1	38	1	0	0	0
Anyplex II HPV 28 (Seegene)	21	L1	15	1	0	0	5	16	3	1	0	1
Hybribio 37 array HPV (Hybribio)	20^c,f^	L1 (MY09/11)	20	0	0	0	0	20	0	0	0	0
HPV-23 Genotyping (Hybribio)	20^c,f^	L1/L2/E1/E2/E4/E6/E7	20	0	0	0	0	20	0	0	0	0
Hybribio 14 HR (Hybribio)	19^c,f^	E6 / E7	19	0	0	0	0	19	0	0	0	0
In-house PCR Luminex	11	L1 / E7	7	0	0	0	4	7	3	1	0	0
In-house realtime PCR	9^e,h^	L1/E1/E4/E6/E7	4	0	0	0	5	4	3	1	0	1
MassArray MALDITOF (Agena)	5^f^	E6 / E7	2	0	3	0	0	5	0	0	0	0
HPV Direct Flow-chip (Master Diagnostica)	5^e,h^	L1 (GP)	4	0	0	0	1	4	0	1	0	0
InnoLiPA Extra (Fujirebio)	5	L1 (SPF10)	2	0	0	0	3	2	2	1	0	0
Anyplex HR HPV (Seegene)	5	L1	3	1	0	0	1	4	0	0	0	0
Real-time PCR MehrViru	4^c,f^	E6/E7/L1/L2	4	0	0	0	0	4	1	0	0	0
In-house PGMY-CHUV	3	L1 (PGMY)	2	0	0	0	1	2	0	0	0	1
HPV SPF10-LiPA25 (Labo-bio)	3^d,g,h^	L1 (SPF10)	0	0	0	0	3	0	0	2	1	0
Cobas 4800 / 6800 (Roche)	3	L1	2	0	0	0	1	2	1	0	0	0
Abbott m2000 / Alinity M (Abbott)	3^f^	L1	0	2	1	0	0	3	0	0	0	0
Tellgen 27plex, 14HR	3^c^	L1 / L2	3	0	0	0	0	3	0	0	0	0
Realquality (AB Analitica)	3^e,h^	E6 / E7	1	0	0	0	2	1	2	0	0	0
In-house NGS	2	L1	1	0	0	0	1	1	1	0	0	0
Onclarity (BD)	2^f^	E6 / E7	1	0	0	1	0	2	0	0	0	0
GenoFlow HPV array (DiagCor)	2^c,f^	L1 (PGMY)	2	0	0	0	0	2	0	0	0	0
Ampliquality (AB Analitica)	2	L1	0	1	0	0	1	1	0	0	0	1
OncoPredict HPV-DNA (Hiantis)	2^c,f^	E6 / E7	2	0	0	0	0	2	0	0	0	0
VisionArray HPV (ZytoVision)	2^f^	L1	0	0	1	1	0	2	0	0	0	0
Other Commercial assays^a^	14	L1/E1/E2/E6/E7	5	0	2	1	6	8	3	0	0	3
Other In-house assays^b^	4	L1 / E6 / E7	1	0	0	1	2	2	0	0	0	2

^a^Other commercial assays include one laboratory using each of: Venus HPV, Harmonia HPV, Molgentix, Papilloplex, AmpFire, OncoPredict Screen, GeneProof, HPV Operon, HPV screen, SACACE HPV screen, yd-diagnostics, aid-diagnostika, Cephid GeneXpert, CLART 4 Genomica.

^b^Other In-house assays include one laboratory using each of: In-house RFLP, In-house Blot, In-house gel-electroforesis and In-house Mass-array.

^c^All datasets provided using the assay were classified as 100% proficient.

^d^All datasets provided using the assay were classified non-proficient.

^e^Most datasets provided using the assay were classified as non-proficient.

^f^All datasets provided using the assay did not report false positivity.

^g^All datasets provided using the assay reported false positivity.

^h^Most datasets provided using the assay reported false positivity.

A total of 182 datasets were obtained using commercially available tests, with the most commonly assays used being Hybribio 21 array (Hybribio, 39 laboratories), Anyplex II HPV 28 (Seegene, 21 laboratories) and 3 other Hybribio assays (20 laboratories) (Table 4). Twenty-eight datasets were generated using assays that either did not discriminate specific HPV types or reported results as HPV 16, 18 and “other” HPV types (Hybribio 14 HR, Roche Cobas 4800/6800 test, Abbot Realtime PCR, High risk HPV Screen, Harmonia HPV, and Oncopredict Screen). These datasets were only analyzed for the specific types tested for individually.

**Table 4:** Proficiency of detecting HPV types and number of false positive results by laboratories that participated in 2021 PP, with data from 2008, 2010, 2011, 2013, 2014, 2017 and 2109 in comparison with all data sets submitted 2021.

		All test by laboratories that participated in 2008, 2010, 2011, 2013, 2014, 2017, 2019 and 2021								All datasets 2021 (%)
		2008 (%)	2010 (%)	2011 (%)	2013 (%)	2014 (%)	2017 (%)	2019 (%)	2021 (%)	
N total participating laboratories / datasets^a^		54/85	98/132	96/134	96/136	121/148	114/141	78/110	132/211	132/211
Overall proficiency^b^		25%	22%	40%	44%	59%	73%	50%	75%	75%
N participating laboratories / datasets^c^		NA	16/27	19/33	21/38	28/43	32/65	43/77	64/94	64/94
Data proficiency comparison*	100% proficient	5 / 17 (29)	5 / 27 (19)	16 / 33 (48)	15 / 38(39)	26 / 43 (60)	35 / 65 (54)	19 / 77 (25)	56 / 94 (60)	158 / 211 (75)
	99–90% proficient	1 / 17 (5.9)	1 / 27 (3.7)	2 / 33 (6.1)	3 / 38 (7.9)	2 / 43 (4.7)	5 / 65 (7.7)	17 / 77 (22)	5 /94 (5.3)	5 / 211 (2.4)
	89–80% proficient	1 / 17 (5.9)	3 / 27 (11)	3 / 33 (9.1)	4 / 38 (11)	3 / 43 (7.0)	6 / 65 (9.2)	6 / 77 (7.8)	2 / 94 (2.1)	7 / 211 (3.3)
	<80% proficient	3 / 17 (18)	3 / 27 (11)	2 / 33 (6.1)	0 / 38 (0)	1 / 43 (2.3)	2 / 65 (3.1)	2 / 77 (2.6)	2 / 94 (2.1)	4 / 211 (0.9)
	Not proficient	7 / 17 (41)	15 / 27 (56)	10 / 33 (30)	16 / 38 (42)	11 / 43 (26)	17 / 65 (26)	33 / 77 (43)	29 / 94 (31)	37 / 211 (17)
False positivity	0 samples	9 / 17 (53)	12 / 27 (44)	23 / 33 (70)	22 / 38 (58)	32 / 43 (74)	48 / 65 (74)	44 / 77 (57)	65 / 94 (69)	174 / 211 (82)
	1 sample	1 / 17 (5.9)	2 / 27 (7.4)	5 / 33 (15)	7 / 38 (18)	2 / 43 (4.7)	8 / 65 (12)	16 / 77 (26)	17 /94 (18)	19 / 211 (9.0)
	2 samples	4 / 17 (24)	5 / 27 (18)	3 / 33 (9.1)	1 / 38 (2.6)	2 / 43 (4.7)	2 / 65 (3.1)	5 / 77 (6.5)	6 / 94 (6.4)	7 / 211 (3.3)
	3 samples	1 / 17 (5.9)	5 / 27 (18)	0 / 33 (0)	3 / 38 (7.9)	3 / 43 (2.3)	1 / 65 (1.5)	6 / 77 (7.8)	1 / 94 (1.1)	1 / 211 (0.5)
	>3 samples	2 / 17 (12)	3 / 27 (11)	2 / 33 (6.1)	5 / 38 (13)	4 / 43 (9.3)	6 / 65 (9.2)	6 / 77 (7.8)	5 / 94 (5.3)	10 / 211 (4.7)

^a^Number of total laboratories and datasets that have been included for each of the corresponding proficiency panels (not considering if they participated other years).

^b^Overall proficiency detected for each proficiency panel not considering if laboratories had participated other years.

^c^Number of total laboratories and datasets laboratories that have participated in the corresponding year as well as in previous proficiency panels. For 2008, the value is non-applicable as it was the first panel study.

*Proficiency detected from datasets belonging to laboratories that had participated in more than one proficiency panel. NA: non-applicable.

Comparison of proficiency and false positivity over the years for laboratories that have participated in at least in two proficiency panels.

Twenty-nine datasets were obtained using a variety of in-house assays.

Overall, 74.88% of datasets (158/211) were 100% proficient and 82.46% (174/211) of datasets showed no false positivity. There were several assays with all the datasets provided showing 100% proficiency (Hybribio 37 array HPV (Hybribio), HPV-23 Genotyping (Hybribio), Hybribio 14 HR (Hybribio), Real-time PCR MehrViru, Tellgen 27plex 14HR, GenoFlow HPV array (DiagCor) and OncoPredict HPV-DNA (Hiantis)), followed by Hybribio 21 array HPV (Hybribio) with 97.44% of datasets being 100% proficient. None of the datasets obtained with HPV SPF10-LiPA25 (Labo-bio), Abbott m2000 / Alinity M (Abbott), Ampliquality (AB Analitica) and VisionArray HPV (ZytoVision) were fully proficient.

Up to 11 assays (all commercial) showed no false positivites, including Abbott m2000 / Alinity M (Abbott), VisionArray HPV (ZytoVision), MassArray MALDITOF (Agena), Onclarity (BD) and Hybribio 37 array HPV (Hybribio), in addition to the assays that were 100% proficient as listed above. Only HPV SPF10-LiPA25 (Labo-bio) showed all datasets (*n* = 3) non-proficient (all of which due to false positivity).

We investigated if there was any specific sample or assay where false positivity was consistently detected. Overall, false positivity appeared to be essentially randomly distributed among samples, indicating that the problem with false positivity was not related to a specific sample nor an assay itself (e.g. cross-reactivity), but rather due to laboratory conditions of use (e.g. cross-contamination).

Comparison of results for laboratories that participated in 2021 as well as in 2008, 2010, 2011, 2013, 2014, 2017 and 2019.

In total, 65 laboratories that participated in 2021 had also participated in at least one of the previous proficiency studies. There were 15 laboratories that submitted results in 2021 that had been participating ever since the first PP was issued in 2008, with 10/15 having participated in all eight previous proficiency studies (2008, 2010, 2011, 2013, 2014, 2017, 2019 and 2021). Some laboratories used the same tests during all years, whereas some laboratories had changed at least one of the tests used.

Comparisons of these results were made for each laboratory using the current proficiency criteria for evaluating datasets obtained during all the years. At the outset in 2008 one false positivity had been allowed, but since 2019 no false positivity is required for 100% proficiency. We now retrieved all crude data from previous years and re-calculated proficiency using exactly the same criteria as used since 2019 (no false positivity allowed).

The proficiency trend for laboratories participating multiple times is shown in Table 3. The 2021 PP revealed an increase in 100% proficiency (from 25% to 60%) and a decrease in <80% proficiency (from 45.1% to 33.1%) when compared to 2019. Moreover, the highest overall proficiency (75% of datasets) was achieved in 2021 – higher than in any previous proficiency study.

## 4 Discussion

We report that the global proficiency in HPV genotyping services had increased in 2021. The increased proficiency is seen both in laboratories that had participated in previous studies as well as in laboratories participating for the first time. This improvement suggests that continuing proficiency testing is helpful to sustain accuracy and to avoid a deterioration in proficiency, as found in 2019. [10]

There are three major strengths of our proficiency study. [10] First, the use of the same PP design as in previous PPs enables a directly comparable estimate of the global development of proficiency in HPV genotyping services. Second, the panel contains all 13 HPV types established as oncogenic and the 2 non-oncogenic vaccine-targeted HPV types, reflecting the HPV genotyping requirements that are most important for HPV vaccine research, surveillance, and monitoring, and third, the broad worldwide distribution (132 laboratories worldwide) and variety of different HPV tests used that make generalizability possible.

Weakness of the study is that the PP was composed for evaluating the HPV genotyping services required to support HPV vaccine research and studies monitoring vaccination impact. However, HPV screening may not need to always separate all different HPV genotypes and the sensitivity requirement may also be different. Similar panels with screening-relevant concentrations of HPV genotypes will be developed to assess and validate the assays (and their use in laboratories) used in cervical screening and triage of high-risk HPV-positive subjects. Another weakness is that we do not have any explanation for the low detectability of HPV68a. Samples were diluted in human DNA as typically found in clinical samples. Further dilution of the samples may have led to a decrease in proficiency. As these samples were readily detectable by a majority of labs, it is unlikely that the low detectability could be caused by a problem with the samples.

The proportion of commercial assays has increased over time, from 57% in 2011, 80% in 2019, and now 86% in 2021 (182/211). Surprisingly, only 13/211 datasets had used any one of the 11 assays that in systematic reviews of the literature had been found to have had published validation data fulfilling all validation criteria (Abbott RealTime High Risk HPV, *n* = 3; Anyplex II HPV HR Detection, *n* = 5; BD Onclarity HPV Assay, *n* = 2; Cobas 4800 HPV Test, *n* = 3). [11] Both Anyplex II HPV HR Detection and Cobas 4800 showed false positivity in one dataset, and none of the 4 other validated assays were fully proficient in more than 2/3ds of datasets. Only 1/3 HPV assays that have been prequalified by WHO was used by any laboratory (Xpert HPV from Cepheid, used by one laboratory). [12]

A common finding, seen also in previous studies, is that the laboratory performing the test has a big impact on the performance of the test itself in particular for certain assays. An example is Anyplex II HPV 28, where 15/21 datasets were fully proficient, but 5/21 datasets were not proficient with 1 to 3 false positive results. An example of assays with robust performance in many laboratories is the assays by Hybribio. These were fully proficient in nearly all datasets (97/98 datasets) from different laboratories, comprising >50% of all fully proficient datasets in the 2021 proficiency study. The widespread use of these assays in laboratories in the WPRO region appears to be a reason why the proficiency was highest in this region. Hybribio was used in 59/132 laboratories, with most of them (44/47) belonging to the WPRO region.

The 2021 Global HPV LabNet HPV DNA proficiency study further enables improving and sustaining sensitivity and specificity of different HPV typing assays. Comparing the results in the 2008, 2010, 2011, 2013, 2014, 2017, 2019 and 2021 global HPV DNA PPs, we can both see that it is possible to achieve a global improvement in proficiency of HPV genotyping services and that regular issuing of PPs is needed for sustaining and improving HPV detection. In the efforts to eliminate cervical cancer, the IHRC will continue to issue PP yearly to promote proficiency in HPV testing. Starting in 2022, we will also be issuing HPV screening panels (with screening-relevant concentrations of the HPV genotypes important for screening) to specifically promote proficiency in HPV screening services.

## Declaration of Competing Interest

The authors declare that they have no known competing financial interests or personal relationships that could have appeared to influence the work reported in this paper
